# Arf GTPase-Activating proteins ADAP1 and ARAP1 regulate incorporation of CD63 in multivesicular bodies

**DOI:** 10.1242/bio.060338

**Published:** 2024-05-10

**Authors:** Kasumi Suzuki, Yoshitaka Okawa, Sharmin Akter, Haruki Ito, Yoko Shiba

**Affiliations:** ^1^ Graduate course of Biological Sciences, Division of Science and Engineering, Graduate School of Arts and Sciences, Iwate University, 020-8551, Morioka, Japan; ^2^ Biological Sciences Course, Faculty of Science and Engineering, Iwate University, 020-8551, Morioka, Japan

**Keywords:** ArfGAP, CD63, Exosome, Extracellular vesicles, Intralumenal vesicles, Multivesicular bodies

## Abstract

Arf GTPase-activating proteins (ArfGAPs) mediate the hydrolysis of GTP bound to ADP-ribosylation factors. ArfGAPs are critical for cargo sorting in the Golgi-to-ER traffic. However, the role of ArfGAPs in sorting into intralumenal vesicles (ILVs) in multivesicular bodies (MVBs) in post-Golgi traffic remains unclear. Exosomes are extracellular vesicles (EVs) of endosomal origin. CD63 is an EV marker. CD63 is enriched ILVs in MVBs of cells. However, the secretion of CD63 positive EVs has not been consistent with the data on CD63 localization in MVBs, and how CD63-containing EVs are formed is yet to be understood. To elucidate the mechanism of CD63 transport to ILVs, we focused on CD63 localization in MVBs and searched for the ArfGAPs involved in CD63 localization. We observed that ADAP1 and ARAP1 depletion inhibited CD63 localization to enlarged endosomes after Rab5Q79L overexpression. We tested epidermal growth factor (EGF) and CD9 localization in MVBs. We observed that ADAP1 and ARAP1 depletion inhibited CD9 localization in enlarged endosomes but not EGF. Our results indicate ADAP1 and ARAP1, regulate incorporation of CD63 and CD9, but not EGF, in overlapped and different MVBs. Our work will contribute to distinguish heterogenous ILVs and exosomes by ArfGAPs.

## Teaser

**Summary:** ADAP1 and ARAP1 regulate CD63 localization in endosomes. ADAP1 and ARAP1 promote incorporation of CD63 and CD9, but not EGF, to multivesicular bodies (MVBs). ADAP1 and ARAP1 can distinguish MVBs with CD63 from MVBs with EGF.

## INTRODUCTION

Arf GTPase-activating proteins (ArfGAPs) mediate the hydrolysis of GTP bound to ADP-ribosylation factors (Arfs), that are small GTP-binding proteins critical for the formation of transport vesicles (Kahn et al., 2008; Donaldson and Jackson, 2011; Sztul et al., 2019). The function of ArfGAPs to hydrolyze Arf-GTP to Arf-GDP had been thought to inhibit the Arf-dependent biological processes. However, increasing evidence indicates that ArfGAP1, the first identified and most well-studied ArfGAP, plays an important role in cargo sorting during the formation of COPI vesicles. ArfGAP1 is required for promoting the process of transport dependent and independent of GAP activity (Shiba et al., 2011; Shiba and Randazzo, 2012; Kahn, 2011; East and Kahn, 2011; Spang et al., 2010). One binding site of COPI to Arf1-GTP binds to the cargo; therefore, the elimination of Arf1-GTP could be required before coat binding to the cargo (Arakel et al., 2019; Dodonova et al., 2017). Consistently, the roles of ArfGAPs have been studied by depleting ArfGAPs rather than overexpressing them to promote GTP hydrolysis on Arf (Shiba et al., 2013; Moshiri et al., 2017; Segeletz et al., 2018; Schoppe et al., 2020; Watanabe et al., 2021).

Extracellular vesicles (EVs) are secreted vesicles that play a role in cell-to-cell communication by transferring their cargos to other cells (Raposo and Stoorvogel, 2013; Mathieu et al., 2019; LeBleu and Kalluri, 2020). Exosomes are EVs of endosomal origin. Early endosomes are formed by the fusion of endocytic and biosynthetic vesicles and mature into multivesicular bodies (MVBs) by inward budding of the limiting membrane to form intralumenal vesicles (ILVs). MVBs fuse with the plasma membrane (PM), and ILVs are secreted as exosomes. CD63 is a tetraspanin protein known to be a marker of exosomes and is enriched in MVBs (Pols and Klumperman, 2009; Stuffers et al., 2009). CD63 forms a complex with other cargo of exosomes, such as Syntenin-1 or LMP1 (Latysheva et al., 2006; Verweij et al., 2011), therefore, it could function as a cargo receptor for exosomes.

The epidermal growth factor (EGF) and its receptor (EGFR) are well-known cargo molecules transported to ILVs (Lill and Sever, 2012; Tomas et al., 2014). When EGF binds to EGFR in the plasma membrane, the EGF/EGFR complex is internalized and transported to the early endosomes. The limiting membrane of early endosomes is inwardly deformed, forming ILVs. ILV formation is mediated by the endosomal sorting complex required for transport (ESCRT) complexes, and the cytoplasmic tail of EGFR, along with the activated signaling complex, is transported into ILVs. MVBs were fused to the lysosomes, and EGF/EGFR, with the signaling complex bound to the cytoplasmic tail of EGFR, was degraded in the lysosomes.

CD63 transport to ILVs may differ from that of the EGF pathway. Quadruple depletion of ESCRT proteins changed the morphology of MVBs, but still formed MVBs where CD63 was localized in ILVs, although EGFR was observed in the limiting membrane (Stuffers et al., 2009). In addition, whether the secretion of CD63 exosome is dependent on ESCRTs is unclear. The depletion of ESCRT proteins often leads to different results: whether the secretion of CD63-positive vesicles is decreased, increased, or remains constant is dependent on different cell lines, protocols, and laboratories (Baietti et al., 2012; Matsui et al., 2021; Colombo et al., 2013).

CD9 has also been a marker of exosomes and observed in CD63-positive vesicles (Matsui et al., 2021; Fordjour et al., 2022). However, CD9 binds and colocalizes with EGFR (Murayama et al., 2008). CD9 localizes to the PM more often than CD63 (Mathieu et al., 2021). Generally, intracellular distribution of CD9 is different than CD63 (Maeda et al., 2023; Mathieu et al., 2021). When CD63 is mutated to localize to the PM similar to CD9, the cells secrete more exosomes; therefore, secretion assays could detect vesicles of PM origin that have a similar size to those of endosomal origin (Fordjour et al., 2022). Furthermore, inhibition of CD63 localization in endosomes promotes the release of exosomes (Larios et al., 2020). The exosome fraction of the secretion assay may include EVs derived from the PM, which are similar in size to EVs of endosomal origin, although not all EVs in the exosome fraction are from the PM. Depletion of Rab27, which is involved in the secretion of secretory granules, decreases the secretion of CD63-positive exosomes (Ostrowski et al., 2010), suggesting that certain exosome fractions in the secretion assay are of endosomal origin. However, how CD63-positive vesicles are formed and whether the mechanism differs from that of CD9, or EGFR are not well understood.

To dissect the CD63 pathway, we focused on CD63 localization. We identified the ArfGAPs that regulate CD63 localization in ILVs using siRNAs targeting ArfGAPs. We used Rab5Q79L to enlarge endosomes. Rab5 is a small G protein that mediates fusion to early endosomes, and its constitutively GTP-bound form mutant, Rab5Q79L, stimulates endosomal fusion, resulting in enlarged endosomes with many ILVs (Stenmark et al., 1994; Wegner et al., 2010). Using ArfGAP siRNAs, we observed that depletion of ADAP1 and ARAP1 inhibited CD63 localization in Rab5-endosomes. We analyzed EGF and CD9 localization in ADAP1 and ARAP1KD cells. Our results indicate that ADAP1 and ARAP1 regulate CD63 transport to ILVs in different population of MVBs.

## RESULTS

### ADAP1 and ARAP1 were determined by siRNA-screening of ArfGAPs in MCF7 cells

To identify the ArfGAPs that regulate CD63 transport to ILVs, we used the human mammary carcinoma cell line MCF7, as we could see clear localization of CD63 inside enlarged endosomes by overexpression of Rab5Q79L. We overexpressed myc-tagged CD63 with CFP-Rab5Q79L, as CD63 localization in Rab5-endosomes was robust. 60-70% of Rab5-endosomes were filled with CD63, and only 30-40% of Rab5 endosomes were without CD63 (Fig. S1B and Fig. 7B, see control).

**Fig. 1.**
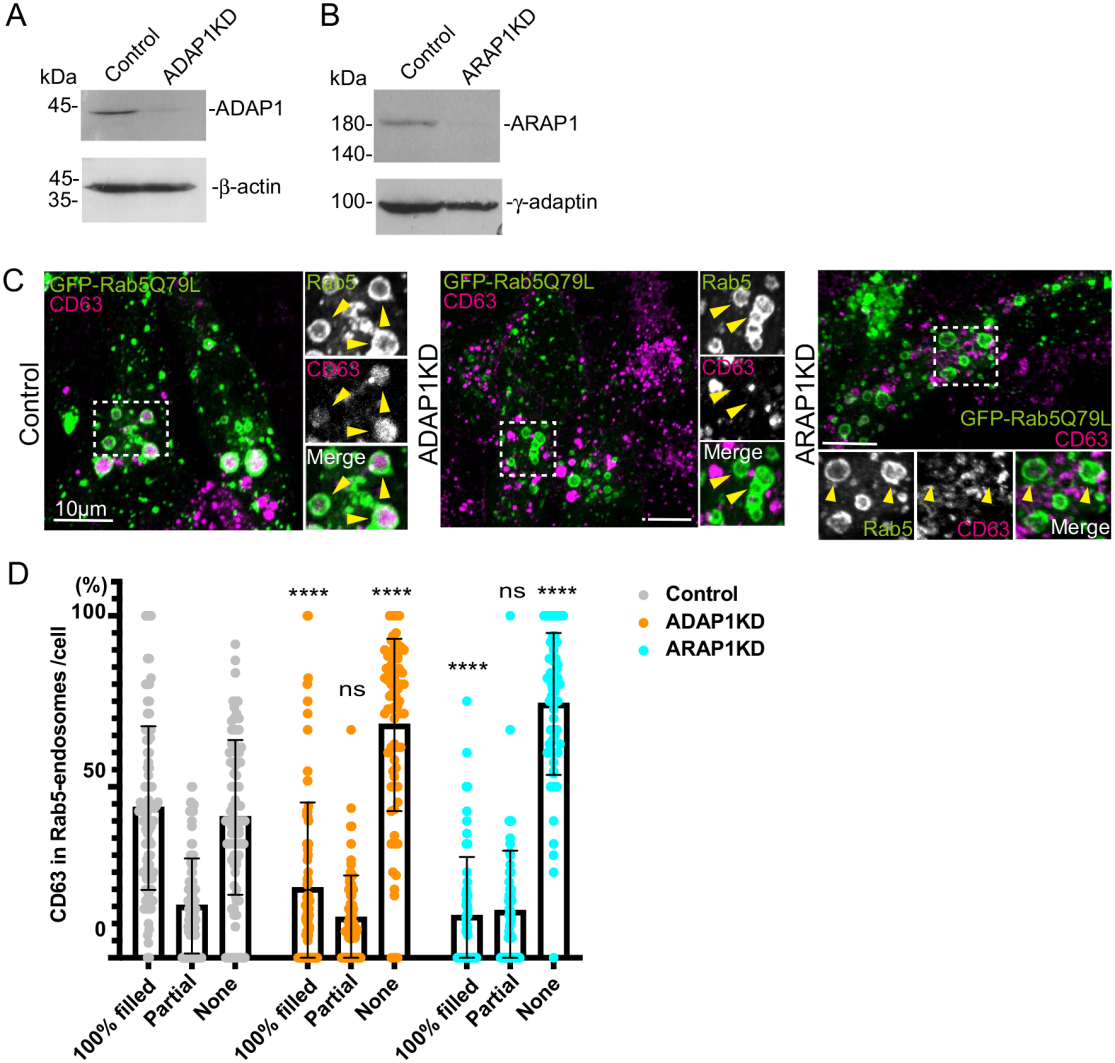
**In ADAP1 and ARAP1KD cells, CD63 incorporation in endosomes is inhibited.** (A) MCF7 cells were transfected with control and ADAP1 siRNAs, and the cell lysates were subjected to western blotting. Note that ADAP1 was efficiently downregulated in ADAP1 siRNA-transfected cells compared with control siRNA-transfected cells. (B) A similar experiment for ARAP1. Note that ARAP1 was efficiently downregulated by its siRNA. (C) The siRNA-transfected cells were transfected with GFP-Rab5Q79L (green) and stained with anti-CD63 (red). The insets were enlarged and Rab5 positive endosomes are indicated with arrowheads. Note that CD63 was filled in endosomes in control cells, whereas CD63 signal was lost in ADAP1 and ARAP1KD cells. (D) The Rab5-positive endosomes were classified 100% filled, partially filled, and none with CD63. The percentage of Rab5 endosomes in each category per cell was shown. More than 15 cells were counted per experiment and the experiment was repeated four times. Error bar, standard deviation (s.d.). Control, *n=*91; ADAP1KD, *n*=73; ARAP1KD, *n=*67. Significance was calculated by two-way ANOVA with Sidak's multiple comparisons test. ns, not significant, ****P<0.0001. Scale bars: 10 µm.

In humans, 31 ArfGAPs are encoded. Of these, AGAP4 siRNA can target AGAP4 to AGAP10 mRNAs; therefore, we used 25 siRNAs against ArfGAPs (Kahn et al., 2008; Shiba et al., 2013). We searched for siRNAs targeting ArfGAPs that decreased CD63 localization in ILVs. In the screening, we classified Rab5-endosomes into two classes, Rab5-endosomes with or without CD63 and calculated their percentages in total Rab5-endosomes per cell (Fig. S1A). Later, we classified Rab5-endosomes in three categories including Rab5-endosomes partially filled with CD63 as ‘partial’ (Fig. 1C,D), but ‘partial’ was only 10-15% in total (Fig. 1D). In the screening, we classified ‘partial’ into Rab5-endosomes without CD63 (Fig. S1A), and compared the percentage of rab5-endosomes without CD63 among ArfGAPs (Fig. S1B). The statistical analyses identified that 13 ArfGAPs affect CD63 localization in Rab5-endosomes (Fig. S1B).

The 13 ArfGAPs may include false positives because of lower expression of CD63-myc or lower number of sample images because of the toxicity by transfection of ArfGAPs siRNAs and CD63-myc/Rab5Q79L. We eliminated 12 ArfGAPs as they clearly showed CD63 localization in Rab5-endosomes, and we repeated the screening for 13 ArfGAPs with staining endogenous CD63. We also included the siRNA of SMCR8, which was recently reported to be an ArfGAP (Su et al., 2021). By staining endogenous CD63, the percentage of Rab5-endosomes filled with CD63 is ∼50%, less efficient than overexpression of CD63 (Fig. S1C, see control). We found GIT1, ADAP1, ARAP1, AGAP11 siRNA-transfected cells increased Rab5-endosomes without CD63 (Fig. S1C). Among them, in ADAP1 and ARAP1-siRNA transfected cells, we could confirm the protein depletion (Fig. 1A,B). Hereafter, we call ADAP1 and ARAP1-siRNA transfected cells as ADAP1 and ARAP1-knockdown (KD) cells. For GIT1, we could not confirm the efficient depletion and eliminate the possibility of off-target effects. For AGAP11, we could not confirm the reproducibility of the results by our hands. In addition, their phenotypes were relatively weak. Therefore, in this manuscript, we focus on ADAP1 and ARAP1.

We performed western blotting and observed that ADAP1 and ARAP1 were depleted by 94.6% and 96.3%, respectively, in siRNA-transfected cells (Fig. 1A,B). To reproduce and analyze our results more detail, we categorized Rab5-endosomes into three classes, i) Rab5-endosomes with 100% filled with CD63, ii) partially filled with CD63, and iii) None (Fig. 1D). We confirmed that in ADAP1 and ARAP1KD cells, the percentage of Rab5-endosomes 100% filled with CD63 were decreased, whereas that of Rab5-endosomes without CD63 (‘None’) were increased. The percentage of Rab5-endosomes partially filled with CD63 did not show the statistically significant difference. (Fig. 1D, Control, i: 43.8%, ii: 15.1%, iii. 41.1%; ADAP1KD, i. 20.3%, *P<*0.0001, ii. 11.6%, iii. 68.1%, *P<*0.0001; ARAP1KD, i. 12.2%, *P<*0.0001, ii. 13.6%, iii. 74.2%, *P<*0.0001).

We tested wild-type (wt) Rab5 expression, however, the GFP-Rab5wt did not induce the enlarged endosomes (Fig. S2A). We examined if CD63 was colocalized with GFP-Rab5wt by Pearson's correlation coefficient (PCC). The PCC value was negative in control cells, indicating CD63 was not colocalized with Rab5wt-endosomes in control condition. We compared the PCC value in ADAP1 and ARAP1KD cells, but the value was not significantly different to that of control cells (Fig. S2B). Rab5Q79L was known to accumulate early and late endosomal cargos in endosomes that have a large number of ILVs (Wegner et al., 2010). By expressing Rab5Q79L mutant, we could analyze the sorting process for ILVs. On the other hand, in Rab5wt expression, CD63 appeared to traffic from Rab5-endosomes without accumulation, and we could not analyze the effect of ADAP1 and ARAP1 depletion.

**Fig. 2.**
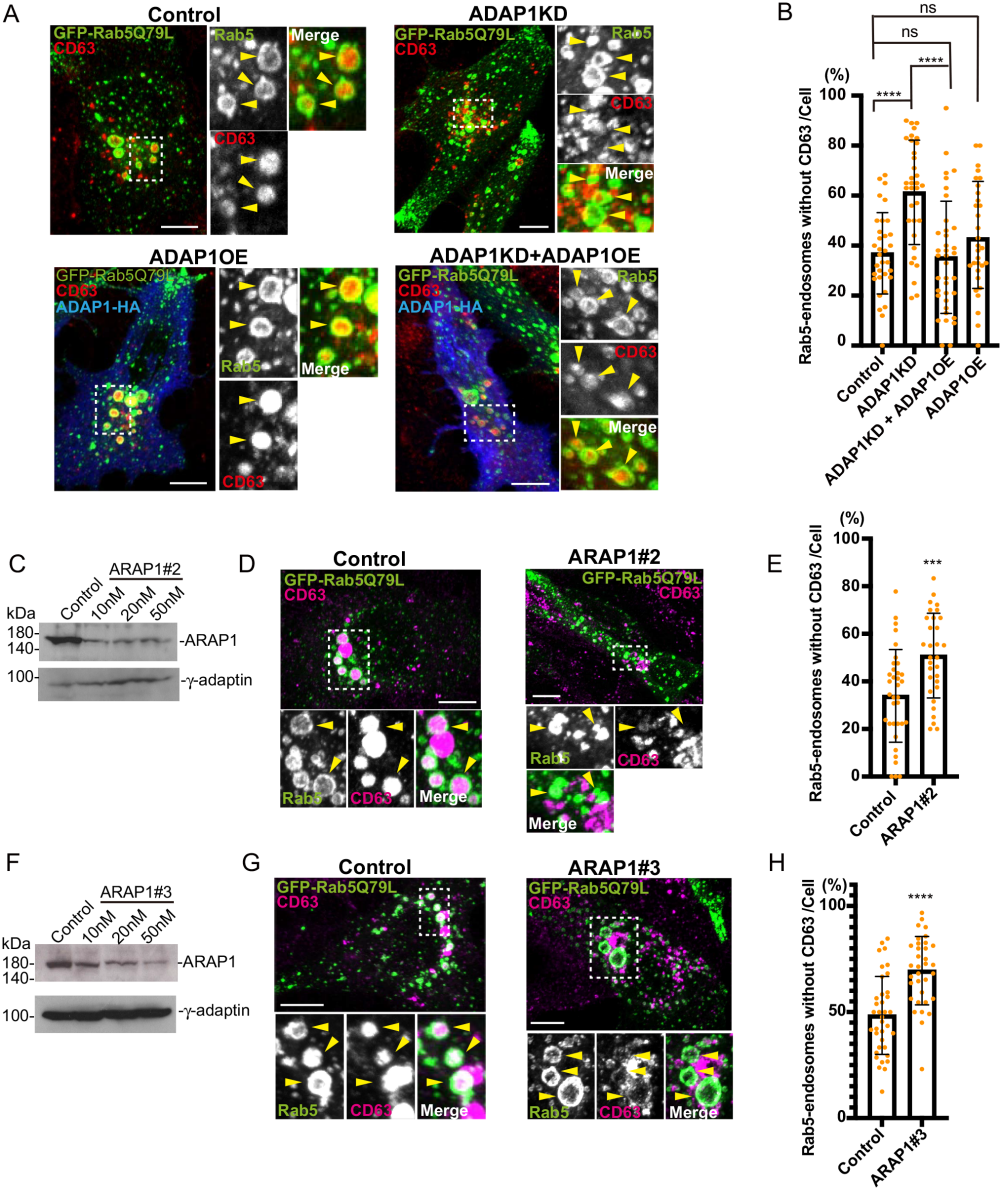
**The increase in endosomes without CD63 in ADAP1 and ARAP1KD cells is not due to off-target effects.** (A) MCF7 transfected control or ADAP1 siRNA were transfected with human ADAP1-HA (ADAP1 overexpression; ADAP1OE, blue) and GFP-Rab5Q79L (green). CD63 was stained with the CD63 antibody (red). The insets were enlarged and Rab5 endosomes are indicated with arrowheads. CD63 signal was seen in the control, while the signal disappeared in ADAP1KD cells. In ADAP1KD+ADAP1OE cells, the CD63 signal in the endosome was recovered. ADAP1OE cells did not show much effect on CD63 in endosomes. (B) The experiment in A was repeated three times. More than 10 cells were counted per experiment and total more than 30 cells were quantified. The percentage of endosomes without CD63 was shown with s.d. Control, *n=*33; ADAP1KD, *n=*34; ADAP1KD+ADAP1OE, *n=*37; ADAP1OE, *n=*31. The Mann–Whitney test was performed. ****P<0.0001; ns, not significant. (C) MCF7 was transfected with siRNA ARAP1 number 2 as indicated and subjected to western blotting. (D) The cells were transfected with control or ARAP1 number 2 siRNA and immunofluorescence was performed as in A. (E) The experiment in D was repeated three times. More than 10 cells were counted per experiment and total more than 30 cells were quantified. Rab5 endosomes without CD63 was shown as in B. Control, *n*=35; ARAP1 number 2, *n*=31. The Mann–Whitney test was performed, **P<0.01. (F) ARAP1 number 3 siRNA was tested for western blotting as in C. (G) Immunofluorescence was performed for control and ARAP1 number 3 cells. (H) The experiment in G was repeated three times. More than 10 cells were counted per experiment and total more than 30 cells were quantified. Rab5 endosomes without CD63 were quantified. Control, *n*=34; ARAP1 number 3; *n*=37. The Mann–Whitney test was performed, ****P<0.0001. Scale bars: 10 µm.

Next, we examined whether the decrease in CD63 localization in endosomes was due to off-target effects. To simplify the results, we categorized Rab5-endosomes into two categories, with or without CD63, similarly to the screening, and compared each sample by the percentage of Rab5-endosomes without CD63 (Fig. 2B,E,H). We transfected wt ADAP1 into ADAP1KD cells to determine whether the phenotype of the ADAP1KD cells was rescued (Fig. 2A). In control cells, CD63 was localized to endosomes, and in ADAP1KD cells, the number of Rab5-endosomes without CD63 increased. When we overexpressed ADAP1 in ADAP1KD cells, CD63 localization in the endosomes was reversed (ADAP1KD+ADAP1OE). Overexpression of ADAP1 in MCF7 cells did not affect CD63 localization in endosomes (ADAP1OE). We quantified endosomes without CD63 and observed that Rab5-endosomes without CD63 were decreased by overexpression of ADAP1 in ADAP1KD cells compared to ADAP1KD cells (Fig. 2B; Control, 36.9%; ADAP1KD, 61.3%; ADAP1KD+ADAP1OE, 35.3%; ADAP1OE, 42.9%). These results confirmed that the decrease in Rab5-endosomes without CD63 in ADAP1KD cells was not an off-target effect.

We performed rescue experiments with ARAP1. However, overexpression of ARAP1 itself increased the number of endosomes without CD63; therefore, we could not observe a rescue of the phenotype by overexpression of ARAP1 in ARAP1KD cells (data not shown). To check the off-target effects of ARAP1 in another way, we analyzed whether two different ARAP1 siRNAs increased the number of endosomes without CD63. Our experiments typically used 10 nM of a mixture of four different siRNAs targeting a single gene. In this experiment, we performed western blotting on the lysates of cells transfected with 10, 20, and 50 nM of individual siRNAs, number 2 and number 3 targeting to ARAP1 (Fig. 2C and F). For number 2 siRNA, we observed a knockdown of 81.8% at 20 nM (Fig. 2C). Therefore, we conducted immunofluorescence using 20 nM number 2 siRNA and observed that CD63 localization in Rab5-endosomes was inhibited compared to that in control cells (Fig. 2D). We quantified Rab5-endosomes and observed that Rab5-endosomes without CD63 increased (Fig. 2E; Control, 33.9%; ARAP1KD number 2, 50.8%, *P<*0.001). Similarly, we performed western blotting for number 3 siRNA of ARAP1 and observed a 78.8% knockdown at 50 nM (Fig. 2F). Immunofluorescence analysis revealed that Rab5-endosomes without CD63 increased (Fig. 2G,H; Control, 48.4%; ARAP1KD number 3, 69.5%, *P<*0.0001). These results confirmed that the perturbations in CD63 localization in Rab5-endosomes of ADAP1 and ARAP1KD cells were not due to off-target effects. By mixing siRNA, we could reduce the toxicity of the siRNA transfection and use a lower concentration of siRNAs (10 nM for the mixture) compared with individual siRNAs (20 nM for number 2 or 50 nM for number 3). In addition, the off-target effects of the mixture are thought to be lower in the single individual siRNA because of lower concentration for each siRNA in the mixture, therefore we used siRNA mixture for other experiments.

### EGF localization in ADAP1 and ARAP1 KD cells

**Fig. 3.**
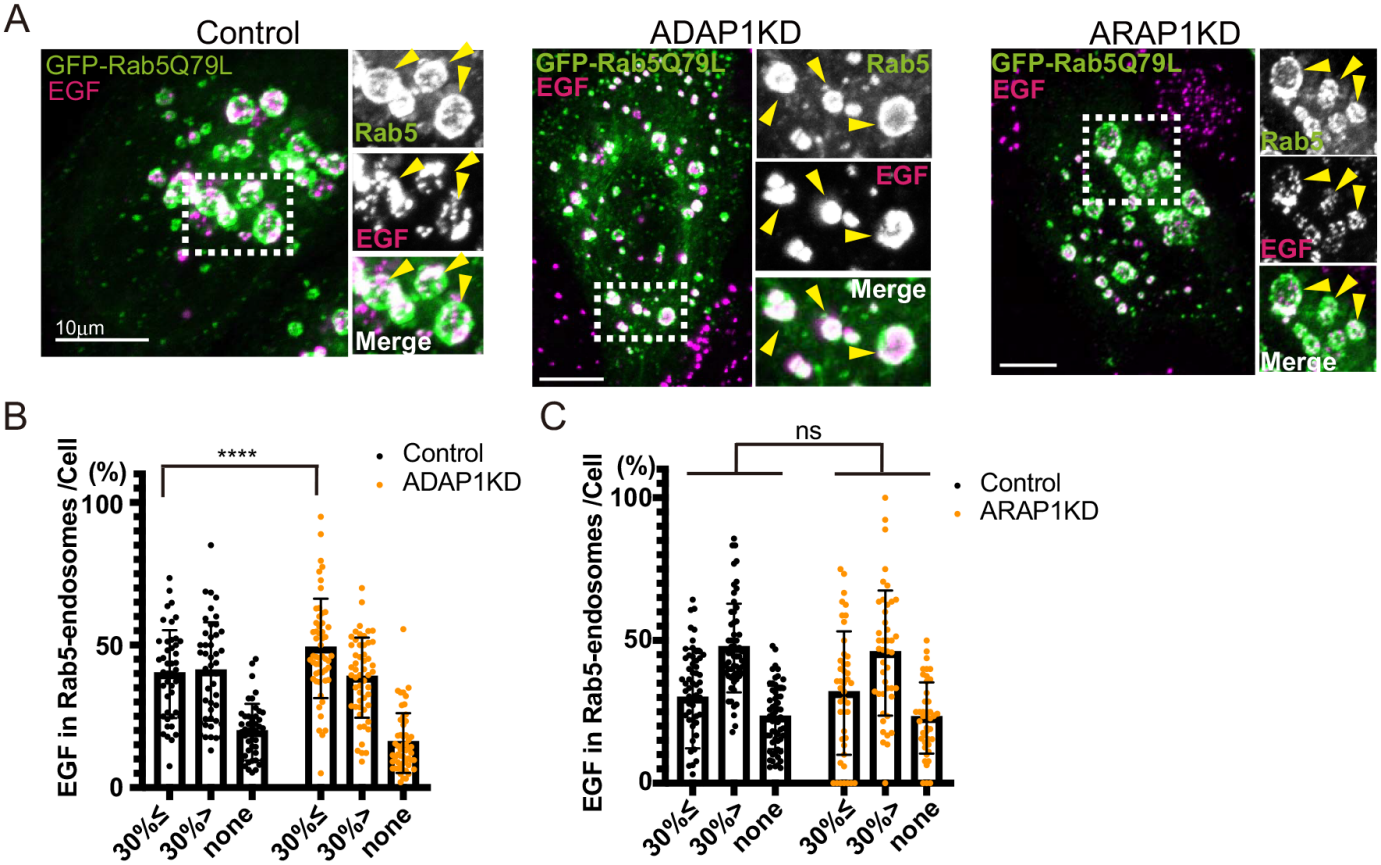
**EGF localization in Rab5 endosomes is not altered in ADAP1 and ARAP1KD cells.** (A) HeLa cells were transfected with siRNAs as indicated. EGF-Alexa 555 was internalized in HeLa cells for 5 min, washed, and incubated for 40 min under leupeptin. The insets were enlarged, and Rab5 endosomes are indicated with arrowheads. Note that EGF in Rab5 endosomes were dotty. (B) The experiment of control and ADAP1KD cells was repeated three times. More than 15 cells were counted per experiment and more than 45 cells were classified as indicated. The percentages of Rab5-endosome per cell are shown with s.d. Control, *n=*46; ADAP1KD, *n=*53. Significance was tested using Mixed-effects analysis. ns, not significant. (C) The experiment of control and ARAP1KD cells was repeated three times. More than 15 cells were counted per experiment and more than 45 cells were classified as indicated. The percentages of Rab5 endosome per cell are shown with s.d. Control, *n=*60: ARAP1KD, *n=*44. Significance was tested using the Mixed-effects analysis. ns, not significant, ****P<0.0001. Scale bars: 10 µm.

To analyze whether EGF localization in Rab5-endosome is affected in ADAP1KD and ARAP1KD cells, we internalized fluorescently labeled EGF for 5 min in HeLa cells, washed them, and incubated them for 40 min to inhibit EGF degradation under Leupeptin. In the case of EGF, its localization in endosomes was usually dotty (Fig. 3A, Control). As CD63 filled 100% in Rab5-endosomes (Fig. 1C, Control), for EGF, we classified the phenotype into three classes: i) endosomes ≧30% filled in; ii) <30% endosomes filled in, which is often EGF on the limiting membranes; and iii) endosomes without EGF signals (none). For ADAP1KD cells, we observed a small increase of Rab5-endosomes ≧30% filled (Fig. 3A,B; Control, i: 39.2%, ii: 40.5%, iii: 20.2%; ADAP1KD, i: 47.2%, *P<*0.0001, ii: 37.2%, iii: 20.1%). For ARAP1KD cells, we did not observe any differences (Fig. 3C, control i: 29.7%, ii: 47.3%, iii: 23.2%: ARAP1KD i: 31.6%, ii: 45.6%, iii: 22.8%). ARAP1 was reported to be involved in EGF internalization and degradation (Yoon et al., 2008; Daniele et al., 2008; Yoon et al., 2011); however, at least for the localization of EGF in Rab5Q79L-overexpressing cells, we did not see the difference in ARAP1KD cells.

Our results indicate that in ADAP1 and ARAP1KD cells, EGF localization in Rab5 endosomes was not inhibited.

### CD9 localization in Rab5 endosomes

**Fig. 4.**
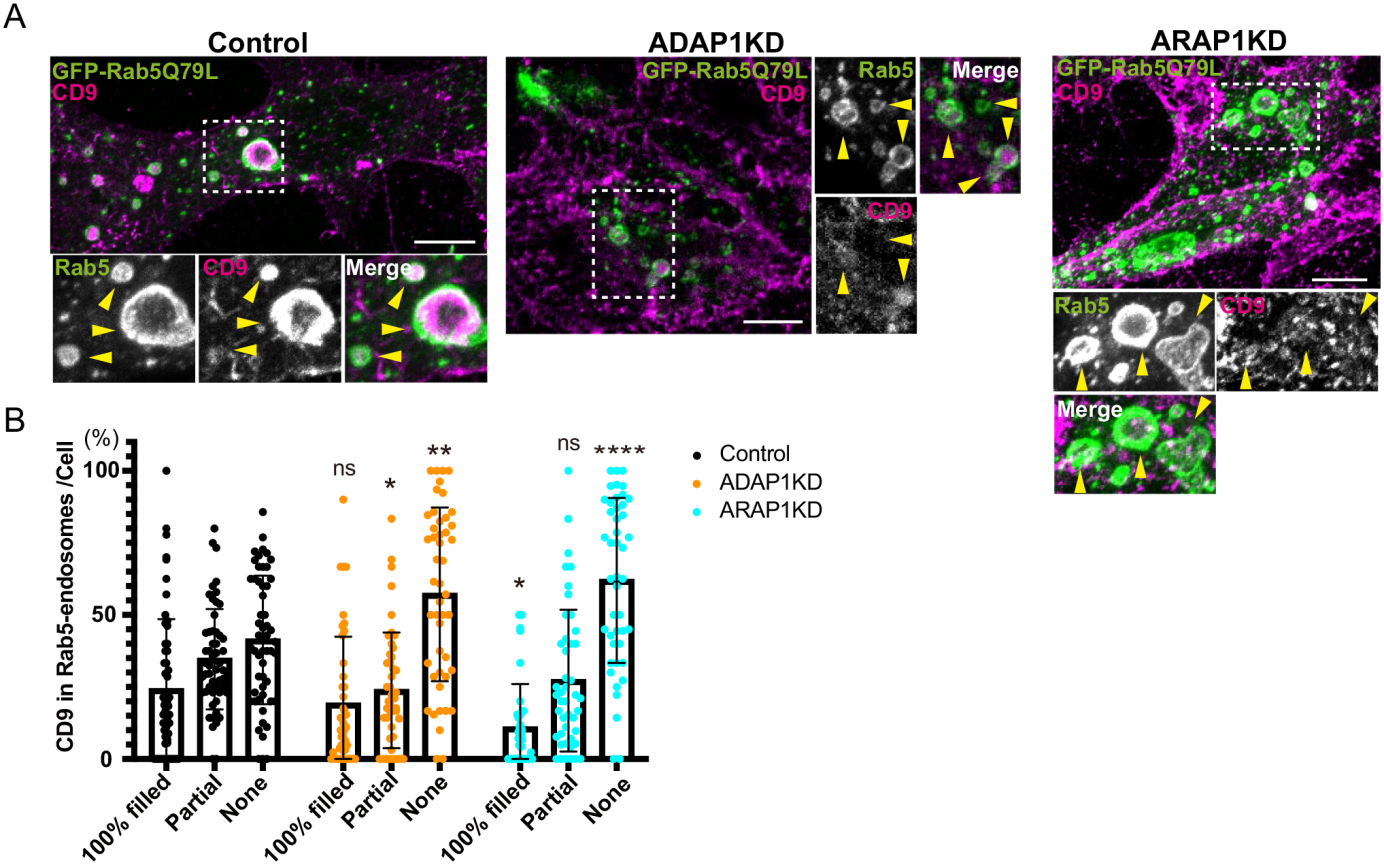
**The incorporation of CD9 in Rab5 endosomes is inhibited in ADAP1 and ARAP1KD cells.** (A) MCF7 cells were transfected with each siRNA as indicated, then transfected with GFP-Rab5Q79L (green), and stained with anti-CD9 antibody (red). The insets were enlarged, and Rab5 endosomes are indicated with arrowheads. CD9 is localized in endosomes in control cells, while in ADAP1 and ARAP1KD cells, CD9 localization in endosomes was decreased. (B) The experiment in A was repeated three times. More than 15 cells were counted per experiment and more than 45 cells were classified as indicated. The percentages of Rab5 endosome per cell are shown with s.d. Control, *n=*55; ADAP1KD, *n=*47; ARAP1KD, *n=*45. Significance was tested using mixed-effects analysis and Sidak's multiple comparison test. *P<0.05, **P<0.01, ***P<0.001, ****P<0.0001, ns, not significant. Scale bars: 10 µm.

Next, we analyzed CD9 localization in ADAP1 and ARAP1KD cells. CD9 showed a more prominent signal in the plasma membrane than CD63, and an endosome signal (Figs 4A,1C). In the case of CD9, many endosomes were filled partially as well as that were filled 100%. Therefore, we classified endosomes into three classes: EGF, i: 100% filled in, ii: partially filled in, and iii: no CD9. We quantified endosomes and calculated the percentage of Rab5 endosomes in each category per cell. We observed that in ADAP1 and ARAP1KD cells, the percentage of Rab5 endosomes without CD9 were increased (Fig. 4B). In ADAP1KD cells, Rab5-endosomes partially filled with CD9 were decreased (Fig. 4B; Control, i: 24.0%, ii: 34.6%, iii: 41.3%; ADAP1KD, i: 19.0%, ii: 23.8%, *P<*0.05; iii: 53.1%, *P<*0.01), whereas in ARAP1KD cells, Rab5 endosomes 100% filled with CD9 were decreased compared with control cells (Fig. 4B; ARAP1KD, i: 10.8%, *P<*0.05, ii: 27.2%, iii: 61.9%, *P<*0.0001). These results suggest that ADAP1 and ARAP1 affect CD9 localization in ILVs differently.

### CD63/CD9/EGF localization into Rab5 endosomes

**Fig. 5.**
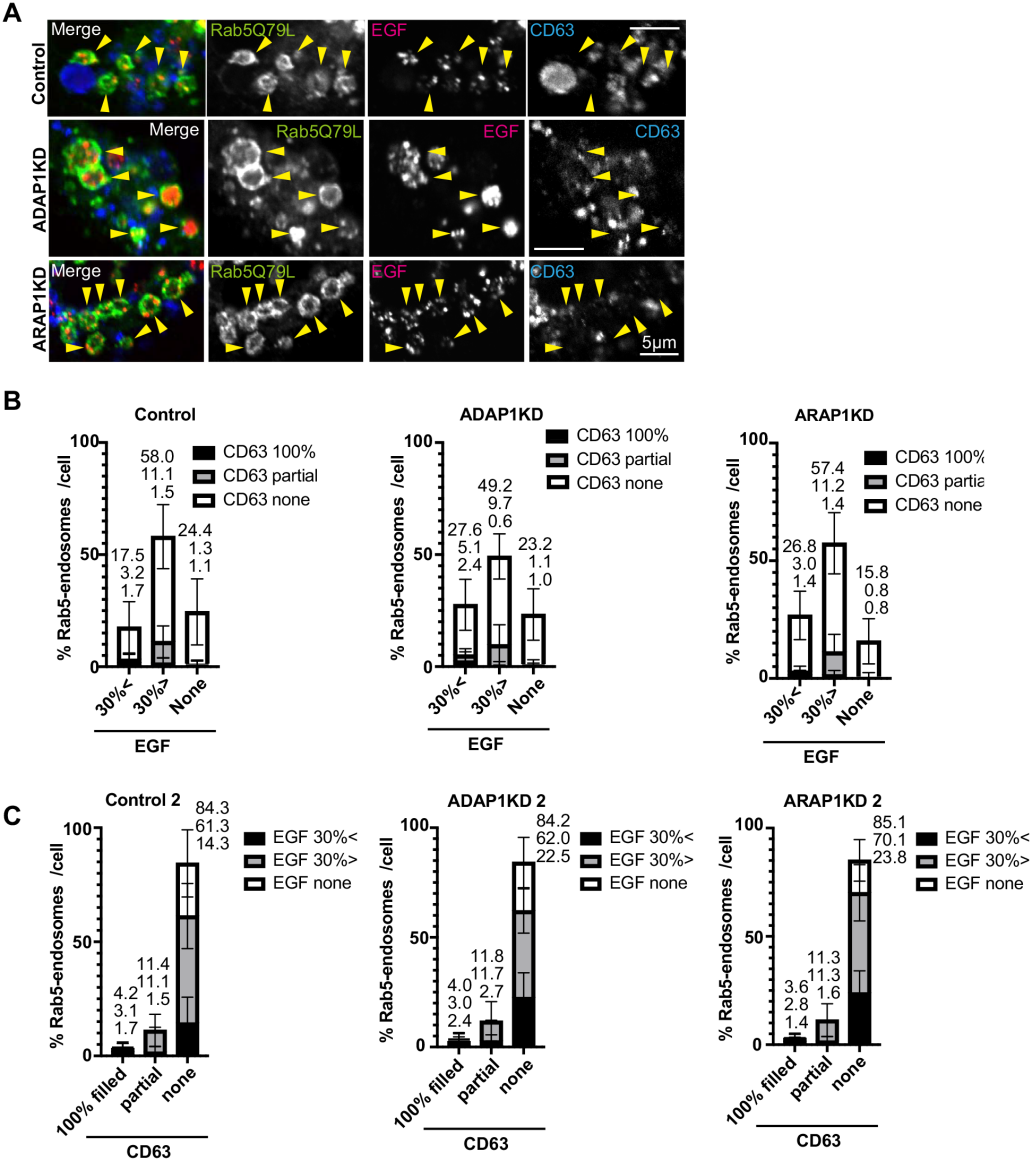
**EGF and CD63 localization in Rab5 endosomes in HeLa cells.** (A) HeLa cells were transfected with each siRNA as indicated, then transfected with GFP-Rab5Q79L (green), and internalized EGF-Alexa 555 for 40 min (red), fixed and stained with anti-CD63 antibody (blue). The representative images of perinuclear region with enlarged endosomes are shown. Rab5 endosomes are indicated with arrowheads. Note that Rab5-endosomes contained dotty signal of EGF, but not CD63. (B) The experiment in A was repeated three times. More than 10 cells were counted per experiment and more than 30 cells were classified as indicated. The percentages of each cell were shown with s.d. The culminated means of each fraction were shown on top of each bar. Control, *n=*32; ADAP1KD, *n=*32; ARAP1KD, *n=*34. (C) The same data set in B were used for the graph of transposed x axis and classification as indicated. Scale bars: 5 µm.

To determine which class of MVBs or ILVs ADAP1 and ARAP1 regulate, we examined MVBs that were positive for CD63 and also positive for CD9 or EGF. For EGF, we used HeLa cells. At baseline, HeLa cells have few Rab5 endosomes containing CD63 (Fig. 5A). We quantified Rab5-endosomes with EGF to determine if these Rab5-endosomes were also positive for CD63 (Fig. 5B,C). As shown in Fig. 5B, EGF positive and negative endosomes both mostly did not have CD63 in HeLa cells. In ADAP1 and ARAP1KD cells, a slight increase (approximately 10%) of Rab5 endosomes >30% EGF was observed (EGF30% <, Control; 17.5%; ADAP1KD; 27.6%; ARAP1KD; 26.8%) and this increase corresponded to the increase of ‘CD63 none’ fraction (EGF30% <, CD63 none, Control; 12.6%; ADAP1KD, 20.1%; ARAP1KD, 22.4%). The same data from Fig. 5B were used for Fig. 5C by transposing x axis and classification. Most Rab5 endosomes were in the ‘CD63 none’ fraction in control cells (CD63 none, Control, 84.3%). We could not see the further inhibition of CD63 ‘100% filled’ or ‘partial’ in ADAP1KD and ARAP1KD cells by HeLa cells. The results indicate that CD63 is excluded from Rab5 endosomes with EGF, and EGF localization into MVBs was not inhibited by ADAP1KD and ARAP1KD cells.

**Fig. 6.**
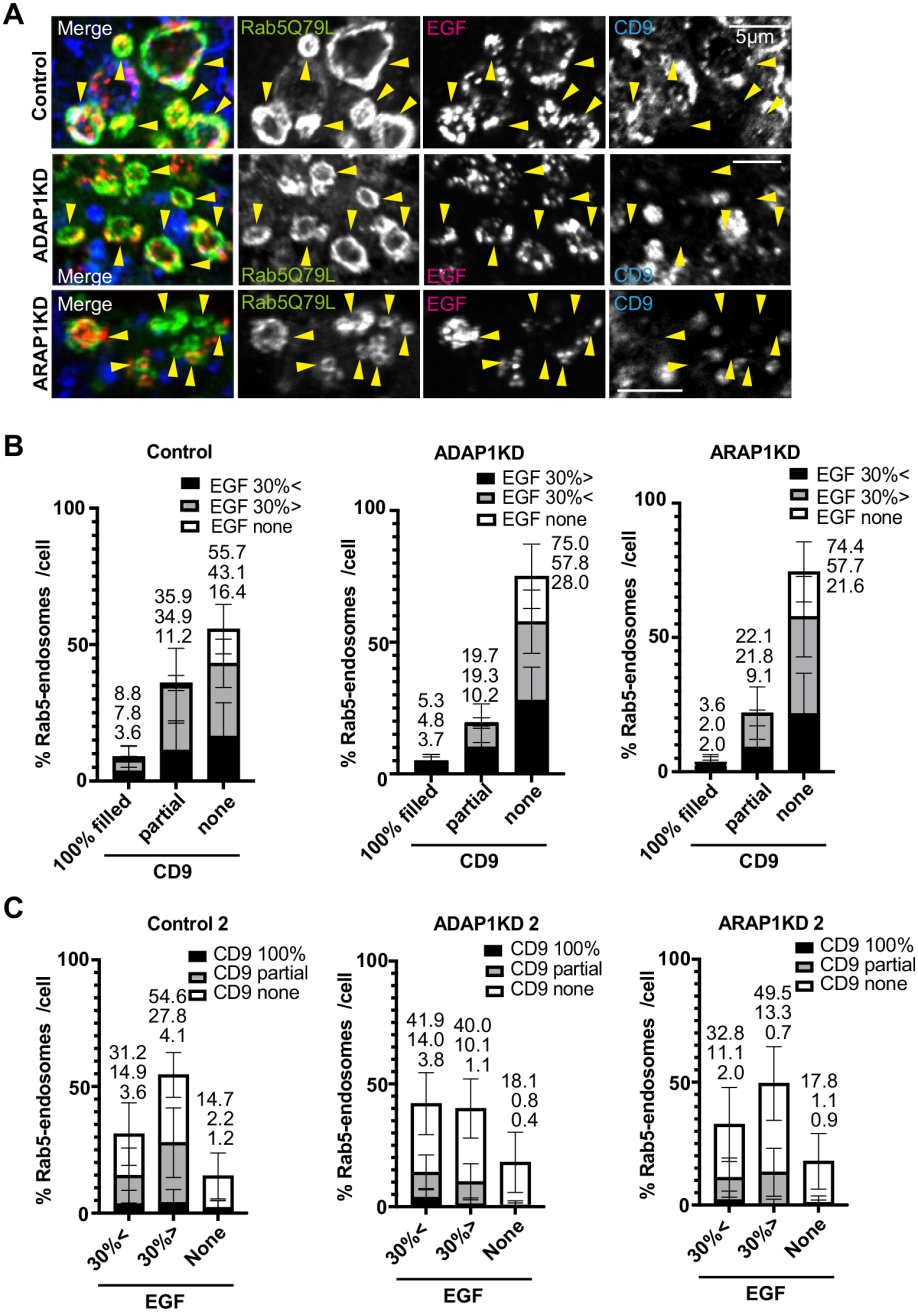
**EGF and CD9 localization in Rab5 endosomes in HeLa cells.** (A) HeLa cells were transfected with each siRNA as indicated, then transfected with GFP-Rab5Q79L (green), and internalized EGF-Alexa 555 for 40 min (red), fixed and stained with anti-CD9 antibody (blue). The representative images of perinuclear region with enlarged endosomes are shown. Rab5 endosomes are indicated with arrowheads. Note that Rab5 endosomes contained dotty signal of EGF as well as CD9 in control cells, but not in ADAP1KD and ARAP1 KD cells. (B) The experiment in A was repeated three times. More than 10 cells were counted per experiment and more than 30 cells were classified as indicated. The percentages of each cell are shown with s.d. The culminated means of each fraction are shown on top of each bar. Control, *n=*39; ADAP1KD, *n=*36; ARAP1KD, *n=*40. (C) The same data in B were used to make the graph of transposed x axis and classification as indicated. Scale bars: 5 µm.

Next, we examined if EGF positive endosomes were also positive for CD9 in HeLa cells (Fig. 6A). We observed Rab5 endosomes that were partially filled with CD9 were also filled with EGF in control cells (CD9 Partial, EGF 30% >; Control, 23.7%; Fig. 6B). In ADAP1KD and ARAP1KD cells, this fraction was decreased (CD9 partial, EGF 30% >; ADAP1KD, 9.1%; ARAP1KD, 12.7%). Instead, Rab5 endosomes without CD9 were increased, and this increase corresponded to the increase of all fractions of EGF (Fig. 6B). In HeLa cells, Rab5 endosomes 100% filled with CD9 were very few, but we could still observe the inhibition in ADAP1KD and ARAP1KD cells (CD9 100%, Control, 8.8%; ADAP1KD, 5.3%; ARAP1KD, 3.6%, Fig. 6B). The transposed data showed that Rab5-endosomes>EGF 30% showed ∼10% increase in ADAP1KD cells again (EGF 30% <, Control, 31.2%; ADAP1KD, 41.9%; ARAP1KD, 32.8%; Fig. 6C). This increased population of Rab5 endosomes was CD9 negative (EGF 30% <, CD9 none, Control, 16.3%; ADAP1KD, 27.9%), suggesting EGF incorporation is independent of CD9 in ADAP1KD cells. These results suggest that Rab5 endosomes partially filled with CD9 was inhibited in ADAP1KD and ARAP1KD cells.

**Fig. 7.**
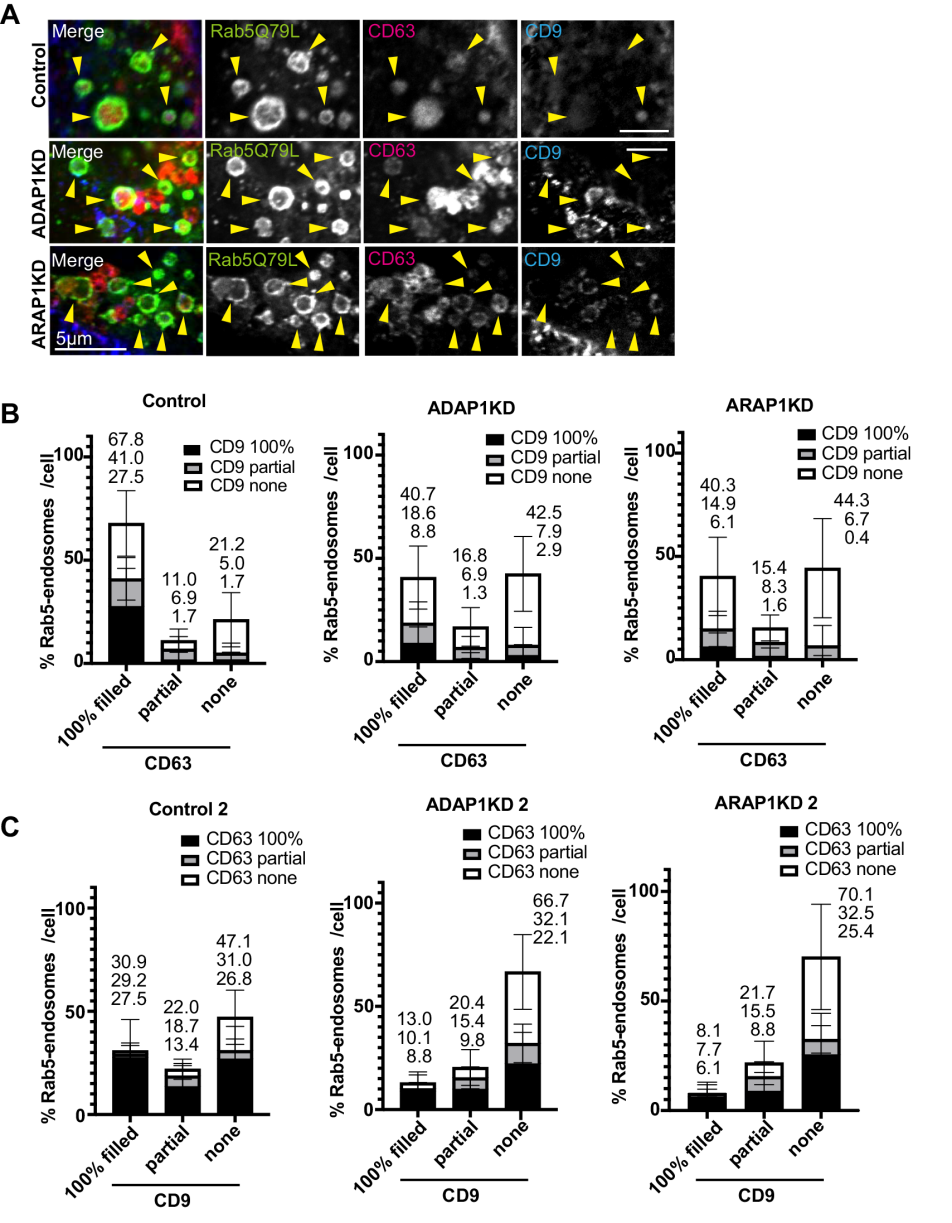
**DsRed-CD63 and CD9 localization in Rab5 endosomes in MCF7 cells.** (A) MCF7 cells were transfected with each siRNA as indicated, then transfected with GFP-Rab5Q79L (green) and DsRed-CD63 (red), fixed and stained with anti-CD9 antibody (blue). The representative images of perinuclear region with enlarged endosomes are shown. Rab5 endosomes are indicated with arrowheads. Note that Rab5 endosomes 100% filled with CD63 also contained CD9 partially or 100%. In ADAP1 and ARAP1KD cells, Rab5 endosomes lost CD63 and CD9 signal. (B) The experiment in A was repeated three times. More than 10 cells were counted per experiment and more than 30 cells were classified as indicated. The percentages of each cell were shown with s.d. The culminated means of each fraction are shown on top of each bar. Control, *n=*40; ADAP1KD, *n=*30; ARAP1KD, *n=*32. (C) The same data in B were used to make the graph of transposed x axis and classification as indicated. Scale bars: 5 µm.

Finally, we examined CD63 and CD9 localization in Rab5 endosmes in MCF7 cells (Fig. 7A). In this experiment, we overexpressed DsRed-CD63 to avoid cross reaction of the mouse antibody to CD9. By overexpression of CD63, the percentage of Rab5-endosomes 100% filled with CD63 was increased, 67.8% in control cells (Fig. 7B). CD9 was often positive for Rab5-endosomes also containing CD63 (CD63 100%, CD9 100%; Control, 27.5%). In ADAP1KD and ARAP1KD cells, the percentage of Rab5 endosomes 100% filled with CD63 and CD9 was decreased (CD63 100%, CD9 100%, ADAP1KD, 8.8%; ARAP1KD, 6.1%), and Rab5 endosomes that have neither CD63 nor CD9 were increased (CD63 none, CD9 none, Control, 16.2%; ADAP1KD, 34.6%; ARAP1KD, 37.6%; Fig. 7B). In the transposed data, the percentage of Rab5-endosomes 100% filled with CD9 were mostly positive for CD63 ‘100% filled’ in control cells and this population was decreased in ADAP1KD and ARAP1KD cells (CD9 100%, CD63 100%, Control, 27.5%; ADAP1KD, 8.8%; ARAP1KD, 6.1%; Fig. 7C). The percentage of Rab5 endosomes without CD63 and CD9 was increased in ADAP1KD and ARAP1KD cells (CD9 none, CD63 none, Control, 16.1%; ADAP1KD, 34.6%; ARAP1KD, 37.6%).

## DISCUSSION

In this study, we identified ADAP1 and ARAP1 as ArfGAPs that promote CD63 localization in endosomes enlarged by Rab5Q79L, that were known to be filled with ILVs (Wegner et al., 2010). We observed that ADAP1 and ARAP1 also affect CD9 localization in MVBs independently to EGF localization.

ARAP1 was previously reported to be involved in EGF transport. In ARAP1 KD cells, EGF internalization was inhibited by FACS analysis (Yoon et al., 2008). Two reports have suggested that EGFR degradation is accelerated in ARAP1KD cells (Yoon et al., 2008, 2011), whereas another report suggested that EGFR degradation is inhibited in ARAP1KD cells (Daniele et al., 2008). Yoon et al. reported in ARAP1KD cells, more EGFR colocalized with EEA1, and Daniele et al. reported that in ARAP1KD cells, more EGFR localized in recycling endosomes (Yoon et al., 2011; Daniele et al., 2008). Although they reported conflicting results, in both cases, ARAP1 is proposed to play a role in EGFR transport from early/recycling endosome to late endosomes. We used Rab5Q79L and the enlarged endosomes by Rab5Q79L were known to have early and late endosome character (Wegner et al., 2010). In addition, we incubated EGF for 40 min, therefore even if there was a delay or acceleration in EGF transport before EGF reached to Rab5 endosomes, EGF could be accumulated in Rab5 endosomes sufficiently. In that case, we might not see the difference on EGF localization in Rab5-endosomes in ARAP1KD cells. Whether ARAP1 functions in two points of EGF transport in early/recycling endosomes and MVBs for CD63 separately, or single point that is connected between early/recycling endosomes for EGF and MVBs for CD63, should be addressed in future study.

EGF and CD63 transport pathways to ILVs have been reported to differ. EGF is known to be dependent on the ESCRT complex, whereas CD63 has an ESCRT-independent pathway to localize to ILVs (Stuffers et al., 2009; van Niel et al., 2011; Edgar et al., 2014). We found ADAP1 and ARAP1 are both involved in CD63 localization in Rab5 endosomes, but the pattern of inhibition is different; we observed that Rab5-endosomes partially filled with CD9 was more inhibited in ADAP1KD cells (Fig. 4B), and a slight increase of Rab5-endosomes with EGF was often observed in ADAP1KD cells (Figs 3B,5B,6C). These results imply that ADAP1 regulates MVBs that are positive for CD9 and EGF both, and ADAP1 depletion inhibited CD9 incorporation, therefore EGF was slightly increased in this class of MVBs. On the other hand, we observed that Rab5 endosomes 100% filled with CD9 was also 100% filled with CD63 and this population was more inhibited in ARAP1KD cells, though ADAP1 also showed the inhibition (Figs 1D,4B,7C). These results suggest that ARAP1 regulates another class of MVBs that were positive for CD63 and CD9 but not EGF. Edgar et al. reported that there are small (<40 nm) and large ILVs (>40 nm). They reported most MVBs contained both small and large ILVs; however, some MVBs contained exclusively small or large ILVs (Edgar et al., 2014). They showed CD63 is the cargo for small ILVs, and EGF for large ILVs. Our results are correlated to their reports when we hypothesized that there are three kinds of MVBs: MVB1 that contains large ILVs with EGF, but not CD63 or CD9. MVB2 that contains both of large and small ILVs with EGF and CD9. MVB3 has small ILVs with CD63 and CD9, which we observed as endosomes 100% filled with CD63 and CD9. MVB1 is independent of ADAP1 and ARAP1. In MVB2, ILVs containing CD9 could be regulated by ADAP1. The slight increase of EGF in ADAP1KD cells imply ILVs of EGF and CD9 are in the same MVBs. Whether ADAP1 regulates small or large ILVs of CD9, will be addressed in future studies. MVB3, which has small ILVs containing CD63 and CD9, is regulated by ARAP1, though ADAP1 could also regulate small ILVs in MVB3. MVB3 does not contain EGF.

Previously we reported that ArfGAP3 is involved in EGF transport from early endosomes to late endosomes (Shiba et al., 2013). It is possible that ArfGAP3 regulates large ILVs in MVB1. To investigate these possibilities more precisely, further analyses will be required by electron microscopy.

ARAP1 exhibits GAP activity against Arf1 and Arf5 dependent on PI (3,4,5) P3 (Miura et al., 2002). In contrast, ADAP1 exhibits GAP activity against Arf6 (Venkateswarlu et al., 2004; Duellberg et al., 2021). Future studies should investigate how Arf and ArfGAP activity of ADAP1 and ARAP1 are involved in CD63 and CD9 sorting into ILVs, and how ADAP1 and ARAP1 regulate exosome secretion. Given the function of ArfGAPs involved in cargo sorting, it is plausible that using different ArfGAPs, we could distinguish the heterogenous ILVs and MVBs. Our work contributes to the understanding of the heterogeneity of MVBs and exosomes.

## MATERIALS AND METHODS

### Reagents

ON-TARGET plus non-targeting siRNA number 4, the siGENOME SMART pool of human ADAP1 and ARAP1, number 2 and number 3 siRNAs of ARAP1, and human ADAP1 and ARAP1 plasmids were purchased from Horizon Discovery Ltd (Cambridge, UK). N-terminal FLAG-tagged human ARAP1 (Miura et al., 2002) and pongo CFP-Rab5Q79L were kindly provided by Dr. Paul Randazzo (NIH, USA), canine GFP-Rab5Q79L and human GFP-Rab5 were from Dr. Marino Zerial (Max Planck Institute of Molecular Cell Biology and Genetics, Germany), and CD63-Myc from Dr. Eiji Morita (Hirosaki University, Japan), respectively. ADAP1 was inserted into pcDNA3.1, with the C-terminally tagged using EcoRI and XhoI. The mouse monoclonal anti-human CD63 antibody (H5C6, DSHB, IA) was kindly provided by Dr. Eiji Morita. The rabbit polyclonal anti-ARAP1 was procured from Abcam (Cambridge, UK), rat monoclonal anti-HA (3F10) from Merck (Darmstadt, Germany), mouse monoclonal anti-myc (9E10) from Invitrogen (Waltham, MA, USA), rabbit polyclonal anti-FLAG from Merck (Darmstadt, Germany), mouse monoclonal anti-β-actin (8H10D10) from Cell Signaling (Danvers, MA, USA), and mouse monoclonal anti-Adaptin γ (88) from BD Biosciences (Franklin Lakes, NJ, USA). Secondary antibodies of goat anti-rabbit IgG conjugated with Alexa Fluor 568 and anti-mouse Alexa conjugated with Fluor 594 and goat anti-mouse IgG, DyLight TM405 Conjugated Highly Cross-adsorbed were purchased from Thermo Fisher Scientific (Waltham, MA, USA), donkey anti-rabbit conjugated with Alexa Fluor^®^ 488 from Abcam, and AffinPure donkey anti-mouse and rabbit IgG conjugated with peroxidase from Jackson ImmunoResearch (West Grove, PA, USA).

### Cell culture

The human mammary carcinoma cell line, MCF7, was purchased from RIKEN BRC (Tsukuba, Japan). MCF7 cells were maintained in DMEM (Nacalai Tesque, Kyoto, Japan) supplemented with 10% FBS (regular; CORNING, NY, USA), 1x MEM Non-essential Amino Acids Solution (Wako, Osaka, Japan), and 1 mmol/l Sodium Pyruvate Solution (Wako). Human cervical cancer HeLa cells were purchased from RIKEN BRC. HeLa cells were maintained in DMEM supplemented with 10% FBS. Cells were typically used by passage six.

### siRNA screening of ArfGAPs in MCF7 cells

A custom cherry-pick siRNA library was constructed from 25 human ArfGAPs and control siRNAs using Horizon Discovery Ltd (Cambridge, UK). We tested several cells and condition of double transfection of siRNAs with Rab5Q79L. We determined to use MCF7 as CD63 can be clearly seen in Rab5-endosomes by overexpression of Rab5Q79L. We used oligofectamin (Thermo Fisher Scientific, MA, USA) to reduce toxicity of siRNA and plasmid transfection. We observed more than 75% knockdown of SMAP1 and ArfGAP3 with western blotting by 200 nM siRNA transfection according to the manufacturer's protocol. After 24 h transfection of siRNA, the medium was changed and CD63-myc and CFP-Rab5Q79L were transfected by Lipofectamin LTX according to the manufacturer's protocol and incubated for another 48 h. The siRNA transfection duration was 72 h. The cells were fixed with 4% PFA/PBS and processed for immunofluorescence analysis. A confocal microscope (Nikon C2, Tokyo, Japan) was used to capture images using 60× objective (NA1.40) and 100× objective (NA 1.45). We captured images of Rab5Q79L-overexpressing cells randomly for each siRNA using the same settings of confocal microscope. More than 15 cells per experiment were analyzed and the experiments were repeated twice. Total more than 30 cells were analyzed except for ADAP1 and ACAP3. We categorized Rab5-endosomes more than 1 μm with or without CD63 by mainly maximum projection images (Fig. S1A). When we could not categorize Rab5-endosomes easily, we inspected 3D slices and determined if CD63 was inside Rab5 endosomes or on the limiting membrane. In the second screening, we used RNAiMax to reduce the amount of siRNA and repeat experiments three times. We tested 10, 20, 50 nM siRNA for ArfGAP1 and observed more than 75% depletion for all concentration. As cells were dead in higher amount of siRNA, we used 10 nM of siRNA for RNAiMAX. After 24 h of siRNA transfection, we transfected GFP-Rab5Q79L by Lipofectamin LTX, and incubated for another 48 h. The siRNA transfection duration was 72 h. We captured images 10-15 cells per experiment and repeated the experiment three times.

### Immunofluorescence

A total of 5×10^4^ MCF7 cells were seeded on coverslips and transfected with siRNAs (10 nM) using Lipofectamine RNAiMAX (Thermo Fisher Scientific, MA, USA) by reverse transfection according to the manufacturer's protocol. The medium was replaced, or cells were transfected with plasmids of GFP-Rab5Q79L 24 h after transfection using Lipofectamine LTX (Thermo Fisher Scientific, MA, USA) according to the manufacturer's protocol. Four hours after the plasmid transfection, the medium was changed. Cells were incubated for 72 h after siRNA transfection.

HeLa cells were used for EGF internalization. After 68 h of siRNA transfection, cells were treated with 100 mg/ml leupeptin (Peptide Institute, Inc. Osaka, Japan). During leupeptin treatment, cells were serum-starved for 1 h, incubated with 100 ng/ml EGF-Alexa 555 (Thermo Fisher Scientific, MA, USA) for 5 min, washed, and incubated at 37°C for 40 min. Treatment with leupeptin was performed for 4 h.

The cells were fixed with 4% paraformaldehyde (PFA) at room temperature for 15 min. After permeabilization and blocking with 0.02% saponin and 0.2% BSA/PBS at room temperature for 30 min, cells were stained with primary antibodies in 0.02% saponin and BSA/PBS for 2 h. After washing three times in 0.02% saponin/0.2% BSA/PBS, the cells were stained with secondary antibodies in PBS for 45 min and washed thrice with PBS. Cells were mounted using Mowiol (Calbiochem, Darmstadt, Germany). We used confocal microscope (Nikon C2 and AX/AXR, Tokyo, Japan) and captured images using 60× objective (NA1.40 and NA1.42) and 100× objective (NA 1.45).

### Image analysis and statistics

For quantification, the same confocal microscopy settings were used for control and ArfGAP siRNA-transfected cells and randomly captured an area of GFP-Rab5Q79L expressing cells. We used the ImageJ Fiji software for image analyses (Schindelin et al., 2012). We counted the number of Rab5Q79L-positive endosomes more than 1 μm manually, and classified Rab5-endosomes as described in the results. ‘100% filled’ means Rab5 endosomes were uniformly filled. ‘Partial’ means part of Rab5 endosomes were filled, often there were bright dotty signals as well as weak signal filled partially in Rab5 endosomes. We calculated the percentage of each category in total Rab5 endosomes per cell. We capture the images of more than 10 cells per experiment and the experiments were repeated three or four times. More than 30 cells were analyzed. For Figs 5-7, to avoid counting the same endosomes several times, we put a transparent sheet on PC and marked each endosome that had been already counted. Statistical analyses were performed using GraphPad PRISM version 10 (GraphPad Software, San Diego, California, USA; www.graphpad.com).

### Western blotting

A total of 5×10^4^ MCF7 cells were transfected with siRNAs using Lipofectamine RNAiMAX. After 72 h of transfection, the cells were washed twice with PBS and lysed with 50 μl of sample buffer (50 mM Tris-HCl pH 6.4, 8% Glycerol, 0.1% bromophenol blue, 50 mM DTT, 2% SDS). An aliquot of 30 μl from each sample was subjected to electrophoresis on 8% or 15% polyacrylamide gel for ARAP1 and ADAP1 each and transferred into an Immobilon^®^-P transfer membrane (Merck Millipore Ltd., Germany). Membranes were blocked with 5% skim milk (Nacalai Tesque, Japan) in Tris-buffered saline containing 0.1% Tween 20 (TBS-T). The membranes were incubated with primary antibodies diluted in Signal Enhancer HIKARI Solution A (Nacalai Tesque, Japan), washed with TBS-T three times, and incubated with horseradish peroxidase-conjugated secondary antibodies for 45 min. Proteins were visualized by enhanced chemiluminescence using an ECL^TM^ western blotting Analysis System (Cytiva, Marlborough, MA, USA). Quantification was performed using the ImageJ Fiji software (Schindelin et al., 2012).

## Supplementary Material

10.1242/biolopen.060338_sup1 Supplementary information
