# Supplementary material for: Arf GTPase-Activating proteins ADAP1 and ARAP1 regulate incorporation of CD63 in multivesicular bodies
Source: Biol Open. 2024 May 10;13(5):bio060338. doi: 10.1242/bio.060338 (PMC11103404; doi:10.1242/bio.060338)
Supplement: Supplementary information [file biolopen-13-060338-s1.pdf]

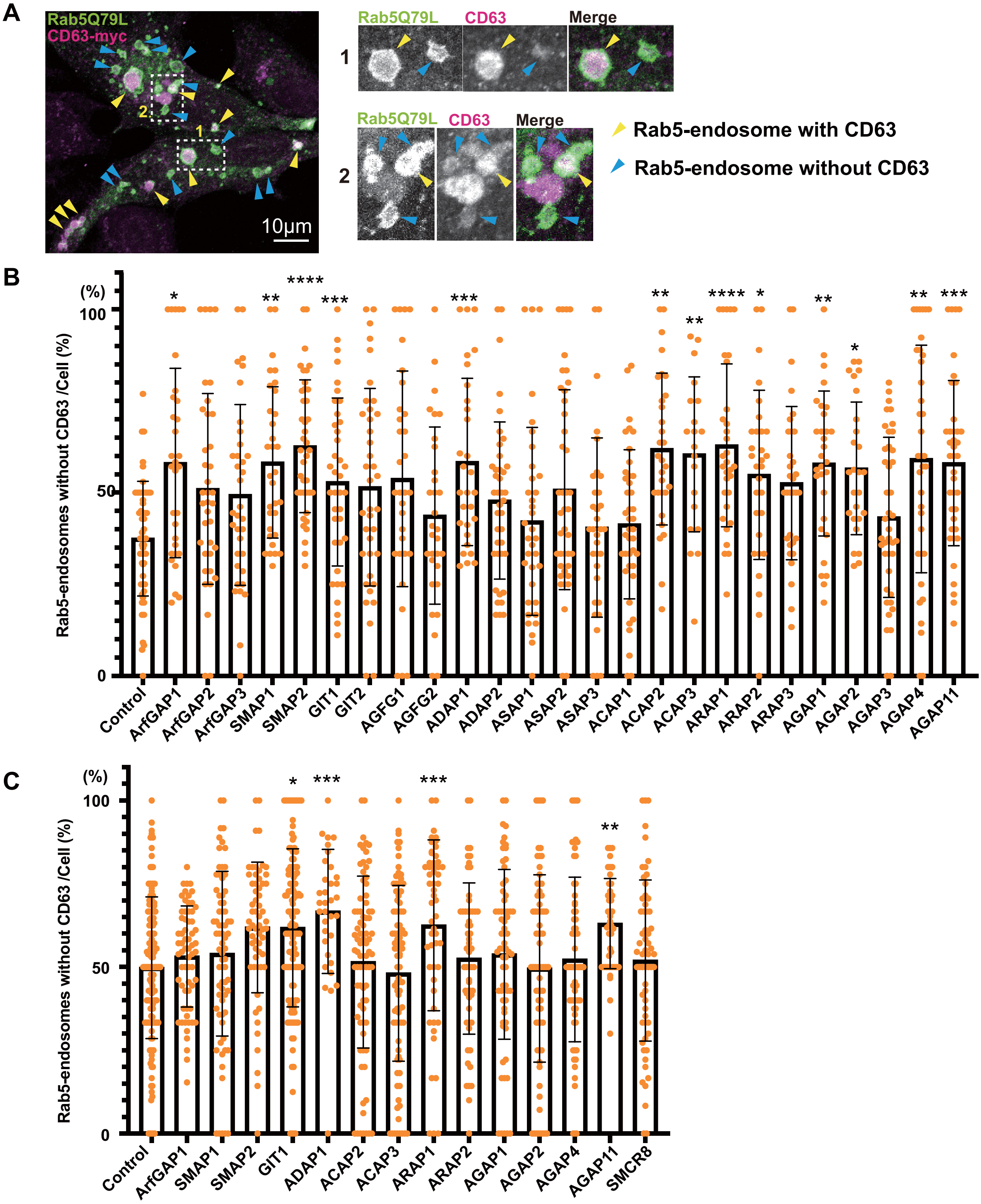

Fig. S1. Screening pf ArfGAPs for CD63 localization.

Biology Open • Supplementary information

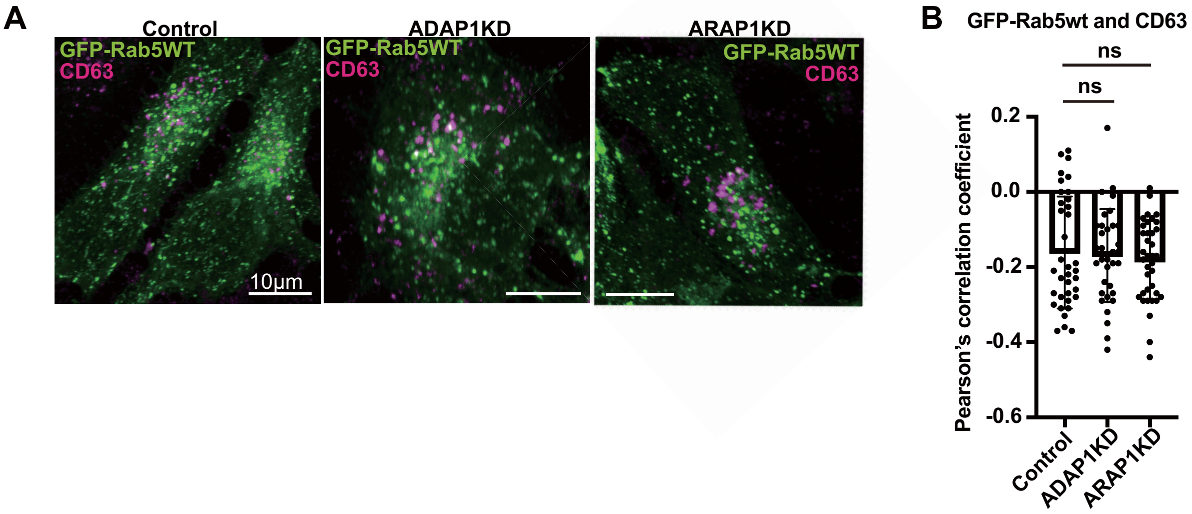

Fig. S2. Rab5WT overexpression does not induce enlarged endosomes.
